# Evaluation of two 4^th^ generation point-of-care assays for the detection of Human Immunodeficiency Virus infection

**DOI:** 10.1371/journal.pone.0183944

**Published:** 2017-08-28

**Authors:** Chrysovalantis Stafylis, Jeffrey D. Klausner

**Affiliations:** Division of Infectious Diseases, Department of Medicine, UCLA David Geffen School of Medicine, Los Angeles, California, United States of America; Waseda University, JAPAN

## Abstract

**Background:**

Fourth generation assays detect simultaneously antibodies for HIV and the p24 antigen, identifying HIV infection earlier than previous generation tests. Previous studies have shown that the Alere Determine HIV-1/2 Combo has lower than anticipated performance in detecting antibodies for HIV and the p24 antigen. Furthermore, there are currently very few studies evaluating the performance of Standard Diagnostics BIOLINE HIV Ag/Ab Combo.

**Objective:**

To evaluate the performance of the Alere Determine HIV-1/2 Combo and the Standard Diagnostics BIOLINE HIV Ag/Ab Combo in a panel of frozen serum samples.

**Study design:**

The testing panel included 133 previously frozen serum specimens from the UCLA Clinical Microbiology & Immunoserology laboratory. Reference testing included testing for HIV antibodies by a 3^rd^ generation enzyme immunoassay followed by HIV RNA detection. Antibody negative and RNA positive sera were also tested by a laboratory 4^th^ generation HIV Ab/Ag enzyme immunoassay.

**Results:**

Reference testing yielded 97 positives for HIV infection and 36 negative samples. Sensitivity of the Alere test was 95% (88–98%), while the SD Bioline sensitivity was 91% (83–96%). Both assays showed 100% (90–100%) specificity. No indeterminate or invalid results were recorded. Among 13 samples with acute infection (HIV RNA positive, HIV antibody negative), 12 were found positive by the first assay and 8 by the second. The antigen component of the Alere assay detected 10 acute samples, while the SD Bioline assay detected only one.

**Conclusions:**

Both rapid assays showed very good overall performance in detecting HIV infection in frozen serum samples, but further improvements are required to improve the performance in acute infection.

## Background

Nearly 1.2 million people in the United States live with Human Immunodeficiency Virus infection, and approximately 14% of them are unaware of their positive status [1]. Early diagnosis and linkage to care is associated with reduced morbidity, mortality and prevention of further transmission [1], thus screening tests that are affordable, accurate and yield results rapidly are important.

Fourth generation testing assays detect simultaneously antibodies for HIV and the p24 antigen. Contrary to the third generation assays, which are limited to antibody detection, the fourth generation assays identify HIV infection early, when HIV antibodies are not yet produced [2]. The addition of p24 antigen detection decreases the window period by 5 days when compared to previous generation tests [3]. Using 4^th^ generation assays for screening high incidence populations has been proven to be more cost-effective compared to 3rd generation tests in identifying new cases, resulting in fewer transmissions and better health outcomes [4]. Additionally, the test format offers the advantage of combining two tests in one assay, thus solving logistic issues in resource-limited settings.

The goal of this study was to evaluate the analytical performance of two rapid 4^th^ generation assays using a panel of serum samples.

## Study design

### Assays under evaluation

The two assays under evaluation were the Alere Determine HIV-1/2 Combo and the Standard Diagnostics BIOLINE HIV Ag/Ab Combo (*SD Combo*). Both are designed to detect HIV-1 and HIV-2 antibodies and the p24 antigen in serum, plasma and whole blood [5, 6]. A comparison of the under evaluation assays is shown on Table 1.

**Table 1:** Characteristics of the under evaluation assays.

	Alere Determine HIV-1/2 Combo^5^	Standard Diagnostics BIOLINE HIV Ag/Ab Combo^7^
**Method**	Lateral Flow	Lateral Flow
**Specimen types**	Serum, plasma, whole blood	Serum, plasma, whole blood
**Antigens to detect anti-HIV**	recombinant gp41 (HIV-1), gp36 (HIV-2)	Not specified
**Time to results**	20 min	20 min
**Instrumentation**	none	none
**Differentiate Ab from Ag**	Yes	Yes
**CLIA status**	Waived	Waived
**FDA status**	Approved	Not Approved
**WHO Prequalification status**	Approved	Approved

### Panel characteristics and testing methodology

The testing panel included 133 frozen serum specimens. The samples were acquired from UCLA Clinical Microbiology & Immunoserology laboratory and were remnants of specimens originally submitted for HIV testing. Positive, negative and acute HIV infection samples were included in the testing panel. All samples were de-identified before being included in the study.

**Fig 1:**
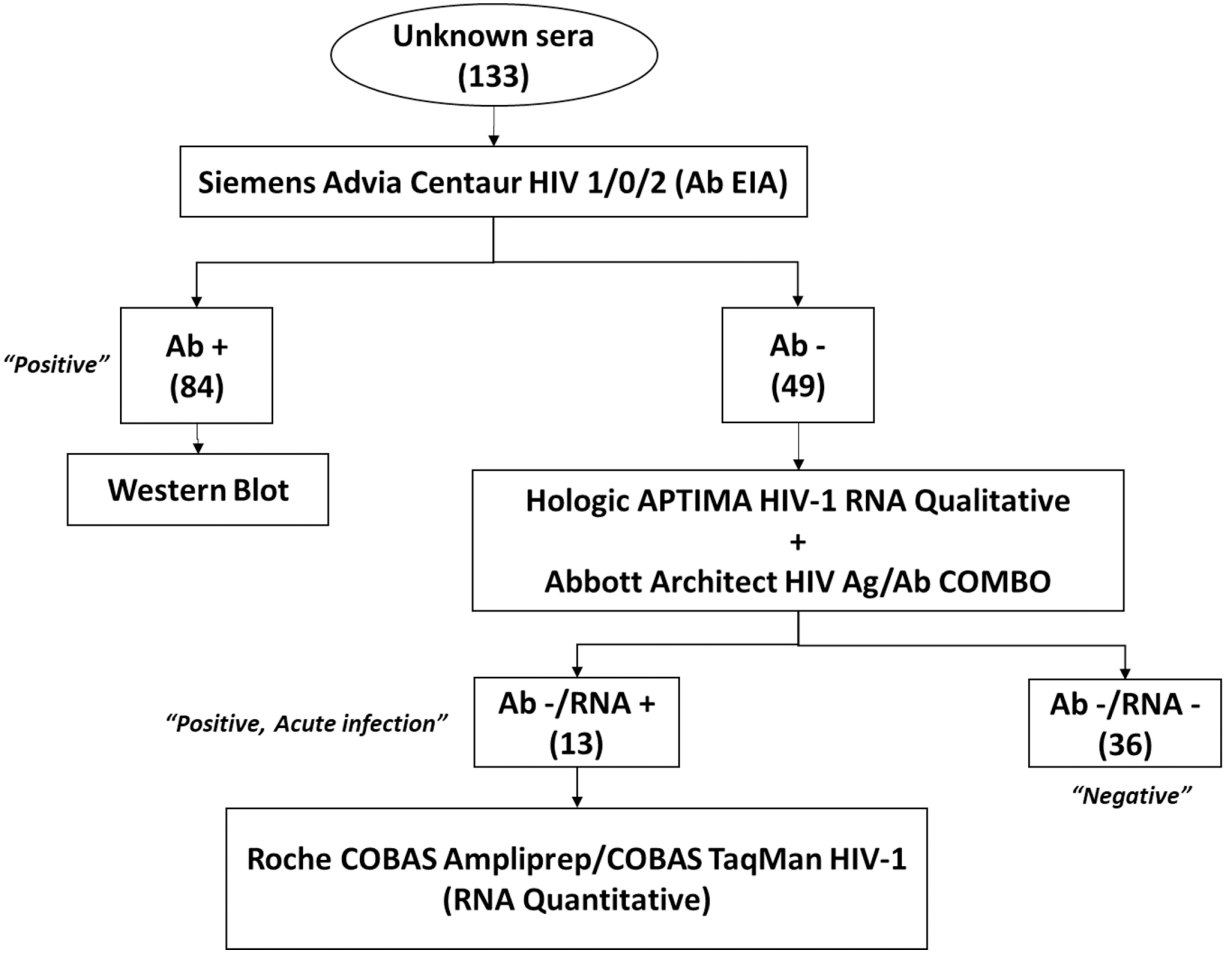
Reference testing algorithm.

The infection status of each sample was confirmed following the testing algorithm shown in Fig 1. Initially, sera were tested for HIV antibodies by the Advia Centaur HIV 1/O/2 enzyme immunoassay (Siemens, Malvern, PA) (Ab EIA). Antibody positive sera were confirmed using the HIV-1 Western Blot. Antibody negative sera were tested for HIV RNA by APTIMA HIV-1 RNA Qualitative test (Hologic, Marlborough, MA), followed by Architect HIV Ag/Ab Combo (Abbott, Illinois, U.S.A). For RNA reactive samples, the COBAS AmpliPrep/COBAS TaqMan HIV-1 v2.0 (Roche, Pleasanton, CA) was used to measure the viral load. A specimen was considered positive for HIV infection, if it tested positive in both Ab EIA and Western Blot or if the Ab EIA was negative and RNA was detected by the GenProbe assay (acute infection). Sera positive by the rapid combo assays were confirmed by Multispot HIV-1/HIV-2. After the reference methods were performed, specimens were stored at -70°C.

Testing and result interpretation were performed in parallel for both assays accordingly to manufacturer’s instructions. Specimens with discordant results were retested by the rapid tests and the reference methods.

### Data analysis

Overall sensitivity and specificity for both devices were calculated based on the results of the reference algorithm. Sensitivity for HIV antibodies was calculated using for reference the results of the Ab EIA. Dara analysis was performed using Stata version 14. The respective 95% confidence interval (95%CI) was calculated using the exact binomial distribution method.

### IRB statement

Approval was provided by UCLA Office of the Human Research Protection Program IRB# 14–000155.

## Results

Reference testing showed that overall 97 specimens were positive for HIV infection and 36 HIV negative. Of the HIV positive samples, thirteen were Ab negative/RNA positive and were classified as “acute infection” samples. The Multispot was reactive only for specimens positive for antibodies and there were no discordant results. Fourteen samples with discordant results (positive according to reference algorithm, but negative according to the rapid assays) were retested, yielding the same results.

The sensitivity for detecting HIV infection among the panel samples was 95% (95%CI 88–98%) for the Alere Determine HIV-1/2 Combo and for the SD Combo 91% (95%CI 83–96%). Both assays showed an overall specificity of 100% (95%CI 90–100%). Both rapid assays showed no false positive results; the Alere Determine HIV-1/2 Combo yielded five false negative results and the SD Combo nine. Finally, no indeterminate or invalid results were recorded.

Sensitivity for detecting HIV antibodies was 95.2% (95%CI 88.3–98.7%) for the Alere Determine HIV-1/2 Combo and 95.2% (95%CI 88.3–98.7%) for the SD Combo. As shown in Table 2, the Architect Combo identified correctly 10 out the 13 HIV antibody negative/ RNA positive samples. Regarding the rapid assays, 12 out of 13 samples were found positive by the Alere Determine HIV-1/2 Combo and only 8 out of 13 were found positive by the SD Combo. The antigen component of the SD Combo reacted weakly in only one sample, while the Alere Determine HIV-1/2 Combo antigen detected 10 samples.

**Table 2:** Test results of “acute” (RNA positive, HIV antibody negative) specimens by Alere and SD combo assays. Comparison with the Abbott Architect HIV Ag/Ab Combo and viral load testing.

Specimen #	Abbott Architect	Alere Determine HIV-1/2 Combo			Standard Diagnostics BIOLINE HIV Ag/Ab Combo			Viral Load (copies/mL)
	Ab/Ag	Ab/Ag	Ag	Ab	Ab/Ag	Ag	Ab	
**121**	-	+	weak +	-	+	-	weak +	316
**122**	-	+	weak +	+	+	-	+	427
**123**	-	+	weak +	-	-	-	-	209
**124**	+	+	weak +	+	+	-	+	831,764
**125**	+	+	weak +	-	_	-	-	589
**126**	+	+	-	+	+	-	+	37,154
**127**	+	+	weak +	+	+	-	+	1,585
**128**	+	+	weak +	+	+	-	+	338,844
**129**	+	+	-	+	+	-	+	91,201
**130**	+	-	-	-	-	-	-	741,310
**131**	+	+	weak +	-	-	-	-	102,329
**132**	+	+	weak +	-	+	weak +	-	2,041,738
**133**	+	+	weak +	-	-	-	-	6,918
**Samples detected**	10/13	12/13	10/13	6/13	8/13	1/13	7/13	

There was no statistical significant difference between the Alere and the SD Bioline test in overall sensitivity (94.8% vs 90.7% respectively, p-value = 0.2671) or in antibody detection performance. On the contrary, there was statistical significant difference in the performance of the antigen components (76.9% vs 7.7% respectively, p-value = 0.001)

## Conclusions

The laboratory performance of two rapid 4^th^ generation assays was evaluated. Both assays performed very well with comparable sensitivity and specificity. There were no false positive results and the frequency of false negative results were low for both kits.

The Alere Determine HIV-1/2 Combo has been previously proven to be superior to 3^rd^ generation assays in detecting HIV antibodies [8,9]. Nevertheless, it showed lower sensitivity in detecting HIV antibodies than the one reported by the manufacturer (99.9%) for serum samples [5]. That finding is consistent with previous studies that have also reported lower performance than anticipated on serum samples [9,10].

Compared to laboratory assays, the Alere Determine HIV-1/2 Combo has shown moderate performance in detecting acute HIV infection [8, 9,10]. Rosenberg et al. (2012) showed that the assay was incapable of detecting p24 antigen (sensitivity = 0.0%) [11] in whole blood samples and other studies using serum samples reported antigen sensitivity ranging from 2.9% to 88% [9,12]. Nevertheless, in our testing panel the p24 component had a sensitivity of 76.9%.

To our knowledge, this is one of the few studies evaluating the SD Combo assay using serum samples. Fransen et al. (2016) studied the performance of the test on 35 acute infection samples and showed that the SD Bioline had sensitivity of 29.4% (95% CI 15.1–47.5) and specificity 100% (89.9–100)[13]. In this evaluation the SD Combo showed also low performance in acute infection samples (sensitivity = 61.5%, 95% CI 31.6%– 86.1%). The overall sensitivity and specificity of the assay were also lower than those reported by the manufacturer (sensitivity = 100%, 95%CI 99.1%-100%; specificity = 99.91%, 95%CI 98%-99.7%)[7], although sample-specific performance is not reported. Further studies with clinical samples are required to evaluate its performance and its potential use as a screening assay.

No formal comparison between these two test kits has been performed. Our analyses showed no difference between the two assays in detecting antibodies for HIV. Significant difference was found in the performance of the antigen component, where the Alere kit identified more acute HIV samples than the Bioline kit. According to package inserts, both lateral flow assays have the same analytical sensitivity of 2 IU/mL in detecting HIV-1 p24 antigen [5,6]. Antigen quantification could be useful in elucidating if this difference is due to technical design or other differences.

A noteworthy finding is that in this evaluation the Architech Combo missed 3 acute samples. Laboratory assays have been shown to perform better that the rapid test kits [14] and show better analytical sensitivity and lower detection limits [15]. Nevertheless, it should be noted that the 4^th^ generation laboratory assay may miss acute infection samples, especially early in infection[14]. As shown on Table 2, the three discordant samples were antibody negative and had a low viral load ranging between 209 to 427 copies/mL, which could be indicative of very early stage acute infection.

Our study had several limitations that need to be taken into consideration. First, a small number of specimens was used to evaluate each assay’s antibody sensitivity and specificity and antigen sensitivity. Additionally, neither the reproducibility nor the variability of the assay was tested. The personnel who performed the testing found the assays easy to use and interpret, but in cases where there was a weak reaction the results were difficult to read. Furthermore, retesting of discordant samples could introduce bias into the study. The findings of our study are limited by the type of sample we selected to use. Despite the rapid tests show better performance in serum samples, using serum requires substantial laboratory infrastructure, which may not be available in resource limited areas.

Fourth generation laboratory assays can detect HIV infection earlier than previous generation assays and they are an integral part of public health effort to diagnose early HIV infection. In this study, we evaluated the performance of two fourth generation rapid test kits using serum samples. Considering the better performance of the rapid test kits in serum compared to whole blood specimens, these kits could be used as a first step in resource-limited settings without substantial laboratory infrastructure. Unfortunately, their current performance limits their role in screening programs. Further research is required to improve the performance of these assays and evaluate their use in screening programs.
